# Mental adjustment to cancer and its relation to anxiety, depression, HRQL and survival in patients with laryngeal cancer - A longitudinal study

**DOI:** 10.1186/1471-2407-11-283

**Published:** 2011-06-30

**Authors:** Mia Johansson, Anna Rydén, Caterina Finizia

**Affiliations:** 1 Department of Otolaryngology - Head and Neck Surgery, Sahlgrenska University Hospital, SE 431 80 Mölndal, Sweden; 2 Astra Zeneca, R&D, SE 431 83 Mölndal, Sweden

## Abstract

**Background:**

Using a longitudinal design, aim of this study was to investigate the relation between mental adjustment to cancer and anxiety, depression, health-related quality of life (HRQL) and survival in patients treated for laryngeal cancer.

**Methods:**

95 patients with Tis-T4 laryngeal cancer were assessed at one and 12 months after start of treatment, respectively, using the Mini-Mental Adjustment to Cancer Scale (Mini-MAC), the European Organisation for Research and Treatment of Cancer (EORTC) Study Group on Quality of Life core questionnaire (EORTC QLQ-C30) supplemented with the Head and Neck cancer module (QLQ-H&N35) and the Hospital Anxiety and Depression (HAD) Scale. For survival analyses patients were followed up for a median time of 4.22 years from inclusion.

**Results:**

The most commonly used adjustment response at both occasions was Fighting Spirit. The use of adjustment responses was relatively stable over time. Correlation analyses showed that patients using Helpless-Hopeless and Anxious Preoccupation responses reported more anxiety and depression, as well as decreased HRQL. Tumour site and stage showed no effect on adjustment response. Survival analysis indicated that use of a Helpless-Hopeless response was related to poorer survival (HR 1.17, p 0.001).

**Conclusion:**

The relation between adjustment responses Helpless-Hopeless and Anxious Preoccupation and anxiety, depression, HRQL and possibly poorer survival indicate that assessment of mental adjustment should be considered when planning treatment and rehabilitation in laryngeal cancer patients.

## Background

To be diagnosed with laryngeal cancer places considerable demand on the patient. Besides the impact of being diagnosed with a life-threatening disease, patients also face psychosocial problems due to impairment in voice and speech and other physical effects caused by treatment [1,2]. As a consequence, laryngeal cancer patients risk mood disorders such as anxiety and depression, as well as decreased health-related quality of life (HRQL) [1,3-5]. Laryngeal cancer patients' different ratings of HRQL and psychological well-being have been associated to both treatment and size of tumour [6]. However, differences in HRQL levels and mental well-being may also be explained by mental coping responses. Over the last decades, there has been a growing interest in coping and, particularly, in the area of coping with cancer. The most widely spread definition of coping is Lazarus' and Folkman's saying coping can be defined as "constantly changing cognitive and behavioural efforts to manage specific external or internal demands that are appraised as taxing or exceeding the resources of a person" [7]. Related to Folkman's and Lazarus' theory of coping is the theory of mental adjustment to cancer, developed by Watson and Greer, where mental adjustment is defined as "the cognitive and behavioural responses the patient makes to the diagnosis of cancer" [8]. Even if the two concepts often are used synonymously, it is argued that there is one predominant difference: the theory of mental adjustment includes emotional reactions to a threatening event, whereas Folkman and Lazarus regard emotional reactions as the outcome of a coping strategy. In this paper we have chosen to use the concept mental adjustment to cancer and, hence, include emotional reactions.

Mental adjustment and coping have been identified as important factors for HRQL and psychological state in cancer patients [8-11]. Adjustment responses such as Fighting Spirit, described as "a highly optimistic attitude, accompanied by a search for greater information about [breast] cancer", have been reported to be beneficial [12-14], whereas responses like Helpless-Hopeless, when patients are devoid of hope and see themselves as gravely ill, have shown a negative impact on HRQL and mental health [14-17]. Furthermore, there is an ongoing debate on the possible impact of mental adjustment on the outcome of cancer, where e.g. Pettingale et al appoint mental adjustment responses to be the single most significant factor in determining both death and recurrence [18]. Studies have demonstrated a negative effect of a Helpless-Hopeless response on five and ten year survival [19,20], whereas e.g. Fighting Spirit has shown to be beneficial for relapse-free survival [18,21,22]. The connection between mental adjustment and survival is however a controversial field and several studies have failed to find any effect of coping or mental adjustment on survival, results further supported by a review article by Petticrew et al [23]. These contradictory findings indicate that the association between mental adjustment and survival in cancer patients is still a matter of interest.

There is a growing interest regarding mental adjustment to cancer in patients with head and neck (H&N) cancer [2,9,24,25], where most studies have indicated an association between an Avoidance response and decreased HRQL [25-27], but also an inverse relation between Fighting Spirit and depression [9]. Due to the multi-factorial and multi-site nature of H&N cancer some authors have expressed the need to investigate adjustment responses in patients with different sites of H&N cancer [24,28]. Furthermore, there has been a demand for longitudinal studies on mental adjustment to cancer [29].

Aim of this study was therefore to investigate longitudinal mental adjustment responses in patients treated for laryngeal cancer and the relation to anxiety, depression, HRQL and survival.

## Methods

### Participants

Study patients were recruited at a weekly tumour conference at Sahlgrenska University Hospital to which all patients with laryngeal cancer in the western part of Sweden are admitted. During the study period 210 patients were admitted to the tumour conference. All eligible patients were consecutively asked to participate. Of the 147 patients judged as eligible, 47 declined participation (22 due to not feeling well enough, three due to family reasons, 22 did not give any reason) while 100 patients accepted to participate. Of the 63 patients deemed not eligible reasons for exclusion were: participation in other studies (19), insufficient knowledge in Swedish language (10), second primary cancer tumour (9), psychiatric disorder (12), dementia (4) and alcohol addiction (9).

All 100 patients included received radiotherapy as part of their treatment. Chemotherapy was given to 9 patients with stage III-IV tumours. One patient was laryngectomised before inclusion, two patients were treated with primary laryngectomy and four patients were treated with laryngectomy as salvage surgery during the study year.

### Study design

This prospective longitudinal study was ongoing between 1998 and 2005 with a discontinuation for two years. Data was collected on six occasions: before start of treatment and at 1, 2, 3, 6 and 12 months after start of treatment. Results presented in this article are based on data collected with Patient Reported Outcome (PRO) instruments at one month and 12 months after start of treatment, respectively. These two measurement points were chosen since they represent quite different situations. One month into treatment a majority of patients are approaching the end of their treatment and suffer from side effects. The experience of receiving the cancer diagnosis is still fresh in memory and the outcome of the disease may still be unclear. Eleven months later the situation is quite different; most patients have recovered well and received reassuring information about their health status. A mail-out/mail-back procedure was used with patients not returning their questionnaires within 2-3 weeks being reminded once. At the tumour conference, diagnosis according to TNM (UICC) and ICD, as well as histopathology, was recorded. Performance status was rated according to the Karnofsky Performance Scale. Patients were also asked about previous and present diseases, present symptoms, weight loss and smoking habits. Socio-demographic data were also recorded.

Two articles based on this material have previously been published [3,30].

### PRO Questionnaires

#### Mini-MAC

The Mini-MAC is a revised version of the widely used Mental Adjustment to Cancer scale (MAC) [8], developed for measuring mental adjustment to cancer in a general cancer population. The Mini-MAC contains 29 items and the psychometric properties of the Mini-MAC have proved satisfactory [31]. The Swedish version of the Mini-MAC has been obtained by standard translation procedures with forward/backward translation, pre-tested on different cancer patients and reviewed by clinicians and patient focus groups. The Mini-MAC items are rated on a 4-point Likert scale ranging from "Definitely does not apply to me" (1) to "Definitely apply to me" (4) and measures patients experiences at present. The Mini-MAC has five domains:

• Helpless-Hopeless, e.g. "I feel completely at a loss about what to do" (8 items)

• Cognitive Avoidance, e.g. "I distract myself when thoughts about my illness cme into my head" (4 items)

• Fighting Spirit, e.g. "I try to fight the illness" (4 items)

• Anxious Preoccupation, e.g. "I worry about the cancer returning or getting worse" (8 items)

• Fatalism, e.g. "I've had a good life; what's left is a bonus" (5 items).

A higher score represents higher endorsement of the adjustment response. The domains can be scored separately through simple addition. Since the domains consist of different number of items we also calculated mean scores by dividing the sum with number of items.

#### HAD

The Hospital Anxiety and Depression (HAD) Scale was developed to detect anxiety and depression in somatically ill patients [32]. The 2-factor structure for anxiety and depression has been confirmed repeatedly [33,34] and the Swedish version has been documented in several studies [35]. The HAD Scale consists of 14 items on a four-point response scale ranging from 0-3. The summary scale scores for anxiety (7 items) and depression (7 items) thus range from 0-21. For both the anxiety and depression factor we have used the cut off values recommended by Zigmond and Snaith [32], where each person is grouped according to a clinically tested classification of psychiatric morbidity where a score < 8 is within the normal range, 8-10 indicates a possible and >10 a probable mood disorder. It has however been discussed whether there is a need for different cut off values for different populations [36,37].

#### EORTC QLQ-C30 and QLQ-H&N35

The European Organisation for Research and Treatment of Cancer (EORTC) Study Group on Quality of Life has developed a modular measurement system for evaluating quality of life in cancer patients [38]. A 30-item core questionnaire, the EORTC QLQ-C30, assesses the physical and psychosocial functioning and symptom experiences of cancer patients in general [39]. To address additional symptoms associated specifically with H&N cancer and its treatment a complementary 35-item module can be used, the QLQ-H&N35 [40,41]. When tested in large, cross-cultural samples of patients with cancer, both the core questionnaire and the H&N cancer-specific module have demonstrated satisfactory to excellent reliability and validity. Calculated scale scores range from 0-100. On the different functioning domains and the Global quality of life a score of 100 corresponds to maximum functioning, whereas on the symptom domains and separate items a score of 100 means worst possible symptoms. For analyses in this paper we used the domains Emotional functioning, Cognitive functioning, Social functioning, Global QoL and Pain. Based on previous research and clinical experience these domains were hypothesized to have the strongest association to mental adjustment.

#### Karnofsky performance scale

Karnofsky index is a physician-completed instrument. It emphasizes physical performance and dependency. Although not designed as a QoL-measure, it is frequently used as one [42]. The index has 11 descriptions ranging from 0% (dead) to 100% (normal) [43].

### Statistical methods

Descriptive statistics were calculated according to standard procedures. Wilcoxon's signed rank test was used for measuring changes over time. Correlations between Mini-Mac domains and domains measuring anxiety, depression and HRQL were calculated using Spearman's correlation. All tests were two-tailed and conducted at 5% significance level. Cases of missing data were handled by imputation, i.e. if less than 50% of items in a domain were missing the calculated mean value replaced missing items. Number of false significances was calculated as ((number of tests-number of significant tests)*alfa)/(1-alfa) [44].

Survival analysis was performed in order to predict death. Possible prognostic variables used were: age, tumour stage, tumour site, previous cancer diagnosis, Karnofsky performance index, family situation and Mini-MAC subscale Helpless-Hopeless, which was used as a continuous score. For ordered categorical or continuous variables Cox's PH-regression was used. Hazard ratios were calculated for descriptive purposes. For multivariate purposes a stepwise Cox's PH-regression was performed. Only variables that affected survival time at univariate tests (p < 0.1) were included as possible predictors in the multivariate analysis.

### Ethical aspects

The study was conducted in accordance with the Declaration of Helsinki and was approved by the ethical committee at Sahlgrenska University Hospital, Gothenburg, Sweden.

## Results

### Participation and compliance

Table 1 contains socio-demographic and clinical characteristics of participants and non-participants. Non-participants differed significantly from participants only in a few aspects: they more often had a supraglottic tumour; advanced disease and worse performance status according to the Karnofsky Performance Scale. Of the 100 participants 5 did not return their questionnaires at evaluation one month after start of treatment, producing a response rate of 95%. At 12 months after start of treatment the response rate was 71%. The 29 drop-out patients missing at 12 months after start of treatment did not differ from the participants completing the study regarding gender, age, civil status or educational level but significantly more were smokers and had a supraglottic localisation.

**Table 1:** Sociodemographic and clinical characteristics of participants and non-participants

	Participants (n = 100)	Non-participants (n = 110)	*p*-value^*†*^
Age, mean years (SD)	67 (11.4)	69 (10.11)	ns
Sex			ns
Female	17 (17%)	22 (20%)	
Male	83 (83%)	88 (80%)	
Tumour site			
Glottic	72 (72%)	61 (55%)	0.0188
Supraglottic	20 (20%)	37 (34%)	0.0382
Subglottic	4 (4%)	3 (3%)	ns
Transglottic	4 (4%)	9 (8%)	ns
Stage			
0	3 (3%)	2 (2%)	
I	57 (57%)	43 (39%)	
II	22 (22%)	24 (22%)	
III	9 (9%)	17 (15%)	
IV	9 (9%)	24 (22%)	0.001
Karnofsky performance scale			
100	60 (63%)	34 (33%)	
90	23 (24%)	33 (31%)	
80	8 (8%)	20 (19%)	
70	3 (3%)	12 (11%)	
60	0 (0%)	1 (1%)	
50	1 (1%)	1 (1%)	
40	0 (0%)	4 (4%)	>0.001
Married/Cohabitant	70 (70%)	62 (56%)	ns
Smokers	50 (50%)	70 (64%)	ns
Loss of weight	21 (21%)	35 (32%)	ns
Residual disease	2 (2%)	2 (2%)	ns
Cardiovascular disease	45 (45%)	38 (35%)	ns
Other malignancy	8 (8%)	11 (10%)	ns

^*†*^p-value significant at ≤0.05, ns = not significant.

Participants were classified as N0M0, except one patient classified as N2M0 and two classified as N2M1. Among non-participants 10 patients were classified as N1M0, six as N2M0, one as N3M0 and one patient as N2M1.

### Mini-MAC

Missing items were low in numbers and psychometric properties according to internal consistency and construct validity were acceptable. Cronbach's alpha for the five domains are displayed in Table 2. Significant positive correlations were found between Anxious Preoccupation and Helpless-Hopeless (ρ = 0.58), Fatalism and Cognitive Avoidance (ρ = 0.49) and Fatalism and Fighting Spirit (ρ = 0.57), calculated at one month after start of treatment. Scores for the different adjustment responses at one and 12 months after start of treatment are displayed in Table 2, together with the observed change between the two measurement points. At both measurement points the most frequently used adjustment response was Fighting Spirit (mean 3.04 and 2.94, respectively), whereas the least used response was Helpless-Hopeless (1.25 and 1.22, respectively). Except for Cognitive Avoidance, all scores decreased during the study but the decrease was statistically significant only for Anxious Preoccupation and Fatalism.

**Table 2:** Mean values, SD and range of scores for Mini-MAC domains one month and 12 months after start of treatment

	Baseline		Follow-up		Change	Baseline
	Mean (SD)	Range	Mean (SD)	Range	*p*-value^†^	Cronbach's alpha
AP total*	14.15 (5.09)	8-29	12.28 (4.07)	8-26	0.01	0.85
AP mean**	1.77 (0.64)	1-3.63	1.54 (0.51)	1-3.25		
CA total*	10.52 (3.34)	4-16	10.94 (3.07)	4-16	ns	0.74
CA mean**	2.63 (0.84)	1-4	2.74 (0.77)	1-4		
FA total*	13.53 (2.99)	5-20	12.87 (3.09)	7-20	0.01	0.61
FA mean**	2.71 (0.60)	1-4	2.57 (0.62)	1.4-4		
FS total*	12.15 (2.58)	4-16	11.76 (2.57)	7-16	ns	0.50
FS mean**	3.04 (0.64)	1-4	2.94 (0.64)	1.75-4		
HH total*	9.97 (3.19)	8-28	9.76 (3.56)	8-5	ns	0.81
HH mean**	1.25 (0.40)	1-3.5	1.22 (0.44)	1-3.13		

AP = Anxious Preoccupation, CA = Cognitive Avoidance, FA = Fatalism, FS = Fighting Spirit, HH = Helpless-Hopeless

*Min-Max scores of AP 8-32; CA 4-16; FA 5-20; FS 4-16;HH 8-32

**Min-Max 1-4 (mean computed by dividing total sum with number of items)

^† ^*p*-value significant at ≤0.05

ns = not significant, Wilcoxon's signed rank test

Number of false significances is calculated to 0.16.

To investigate the possible effect of tumour site and stage on mental adjustment patients were grouped into glottic vs. supra-/sub-/transglottic and stages cis - II vs. III - IV. No significant differences regarding level of response were found between patients with different tumour sites or stages (data not shown).

### Relationships between mental adjustment, anxiety, depression and HRQL

Correlations between the Mini-MAC and pre-specified domains from the HAD, EORTC, and age are displayed in Table 3. The highest correlations at both measurement points were found between the Anxious Preoccupation and Helpless-Hopeless responses and Anxiety, Depression and Emotional Functioning. Although weaker, significant correlations were also found between these adjustment responses and other EORTC domains. Furthermore, when analysing adjustment response by HAD subgroups, results showed that patients with probable or possible mood disorders reported significantly increased scores for Helpless-Hopeless and Anxious Preoccupation responses (data not shown). For the Cognitive Avoidance, Fatalism and Fighting Spirit responses only weak or no correlations were found.

**Table 3:** Mini-MAC correlations with HADS, EORTC QLQ-C30, EORTC QLQ-H&N35 and age, one month and 12 months after start of treatment

	Anxious Preoccupation	Cognitive Avoidance	Fatalism	Fighting Spirit	Helpless-Hopeless
**HAD - Anxiety**					
1 month	0.66 (<0.0001)	-0.11	-0.12	-0.17	0.50 (<0.0001)
12 months	0.59 (<0.0001)	-0.16	-0.11	-0.36 (0.002)	0.58 (<0.0001)
**HAD-Depression**					
1 month	0.56 (<0.0001)	-0.32	-0.09	-0.14	0.52 (<0.0001)
12 months	0.58 (<0.0001)	-0.14	-0.06	-0.20	0.62 (<0.0001)
**EORTC QLQ-C30**					
**Emotional functioning**					
1 month	-0.63 (<0.0001)	0.02	0.02	0.11	-0.46 (<0.0001)
12 months	-0.52 (<0.0001)	0.10	0.14	0.31 (0.008)	-0.49 (<0.0001)
**Cognitive functioning**					
1 month	-0.39 (<0.0001)	0.01	0.13	0.13	-0.34 (0.0007)
12 months	-0.45 (<0.0001)	0.06	0.16	0.35 (0.003)	-0.30 (0.01)
**Social functioning**					
1 month	-0.36 (0.0003)	0.09	0.02	-0.03	-0.34 (0.0007)
12 months	-0.44 (0.0001)	0.17	0.18	-0.08	-0.33 (0.004)
**Global QoL**					
1 month	-0.39 (<0.0001)	-0.035	-0.03	-0.03	-0.30 (0.003)
12 months	-0.56 (<0.0001)	-0.12	0.15	-0.37 (0.002)	-0.53 (<0.0001)
**Pain**					
1 month	0.41 (<0.0001)	-0.06	0.13	0.08	0.36 (0.0004)
12 months	0.28 (0.018)	-0.15	-0.03	-0.15	0.24 (0.039)

Spearman correlation coefficients (significant *p*-value).

Number of false significances is calculated to 2.

### Survival analysis

For survival analyses patients were followed up via medical journals and the Swedish national registration. Median time for follow up was 4.22 years from inclusion (inter-quartile-range 2.34 - 7.58 years). During this period 36 patients passed away. No patient was lost to follow-up. Table 4 contains the results of the univariate survival analysis and Table 5 the results of the multivariate survival analysis. The results indicate that the risk of death in patients with high scores on the Mini-MAC Helpless-Hopeless scale was slightly increased. Remaining scales of the Mini-MAC showed no significant effect on survival. There was no evidence of an increasing or decreasing trend over time in the Hazard ratio.

**Table 4:** Univariate survival analysis

Variable	Hazard Ratio (95% CI)	Cox *p*-value
**Age**	1.049 (1.013-1.086)	0.007
**Stage**		
cis+I+II *vs. *III+IV	2.082 (1.008-4.301)	0.048
**Family situation**		
married/cohabitant *vs. *others	0.501 (0.262-0.957)	0.036
**Tumour site**		
Glottic *vs. *others	0.315 (0.166-0.597)	<0.001
**Karnofsky Performance scale**	0.949 (0.921-0.977)	0.001
**Previous cancer diagnosis**	2.727 (1.060-7.013)	0.037
**Mini-MAC - Helpless-Hopeless**	1.168 (1.067-1.279)	0.001

Number of false significances is calculated to 0.

**Table 5:** Multivariate survival analysis

Variable	Hazard Ratio (95% CI)
**Age**	1.065 (1.02-1.11)
**Stage**	
cis+I+II *vs. *III+IV	1.149 (0.44-3.00)
**Tumour site**	
glottic *vs. *others	0.315 (0.13-0.74)
**Mini-MAC-Helpless-Hopeless**	1.234 (1.12-1.36)

## Discussion

Using a longitudinal design, aim of this study was to investigate the relation between mental adjustment to cancer and anxiety, depression, HRQL, and survival in patients treated for laryngeal cancer. At both measurement points a higher score on the Helpless-Hopeless and Anxious Preoccupation domain was associated with anxiety, depression and decreased HRQL. These findings are in line with what has previously been demonstrated in other cancer diagnoses [14,17]. Tumour site and stage showed no effect on adjustment response. However, survival analysis suggested a slightly increased risk of death for patients using a Helpless-Hopeless response. Data presented in this article was gathered at two occasions and the measurement points were chosen because they represent quite different situations. At one month after start of treatment a majority of patients are at the end of their treatment and many suffer from side effects. Furthermore, the experience of receiving the cancer diagnosis is fresh in memory and the outcome of the disease might still be unclear. Eleven months later the situation is quite different; most patients have recovered well and received reassuring information about their health status. According to Lazarus' and Folkman's transactional theory [7] the use of coping is motivated by how we cognitively appraise a situation and it could be assumed that these patients would appraise their situation as less stressful at the latter measurement point. Hence, it could be expected that the pattern of adjustment responses should change over time with, in particular, a decreased use of both Anxious Preoccupation and Helpless-Hopeless responses. However, of the two, only Anxious Preoccupation obtained statistical significance while Helpless-Hopeless, together with Fighting Spirit and Cognitive Avoidance, remained stable. This impression of stability has previously been demonstrated by Nordin et al [15] and it can be argued that it reflects an aspect of personality [15,45] or life experience given the homogeneity in age and gender for this group. Regarding the Helpless-Hopeless response, the tendency for a floor effect could also leave little room for improvement. A statistically significant decrease was seen for the Fatalism dimension, as previously described also in patients with gastrointestinal cancer [15].

Since the transactional theory of coping emphasizes the importance of the context, one could expect more severe stressors, such as more advanced disease, to be associated with more extensive coping efforts or adjustment responses. This has been indicated by previous research, e.g. Epping-Jordan et al, who showed more advanced stages of breast cancer to be connected to greater use of disengagement coping strategies [46]. In our material however, no significant differences regarding level of response were found between patients with different tumour sites or stages. This lack of association could be due to the fact that a majority of participants had glottic tumours and less advanced tumour stages.

The positive correlations observed between Anxious Preoccupation and Helpless-Hopeless and both anxiety and depression are in line with findings in previous studies [15,17,31]. The relation between these adjustment responses and psychiatric morbidity was even more transparent when analysis demonstrated that the use of both Helpless-Hopeless and Anxious Preoccupation was significantly more common among patients with a possible/probable mood disorder. This relation seems to be stable over time; results of correlation analyses at one and 12 months are more or less unchanged, demonstrating that a Helpless-Hopeless or an Anxious Preoccupation response has a negative impact on well-being, irrespective of phase of illness.

Anxious Preoccupation and Helpless-Hopeless were also associated with pain, a finding previously demonstrated by Okano et al [47]. The causal relation is not evident - does untreated pain increase patients' use of maladaptive adjustment responses or does the use of these adjustment responses make patients more sensitive to pain? In either case, it could indicate the importance of proper analgesic treatment and the possibility to reduce the sensation of pain if the use of maladaptive adjustment responses could be modified.

Fighting Spirit is frequently claimed to have a beneficial effect on psychiatric morbidity [14,48]. Although being the most frequently reported adjustment response by our patients, we found no significant correlation between Fighting Spirit and mood disorders at the first measurement point, a finding also demonstrated by e.g. Grassi et al [17]. However, at follow-up 12 months after start of treatment a higher score on the Fighting Spirit subscale was associated to lower levels of anxiety. This could indicate that a Fighting Spirit response may facilitate the ability to move on with life beyond the cancer. A seemingly illogical finding was the negative correlation between Fighting Spirit and Global Quality of life at 12 months. It should however be noted that Global Quality of life measured by the EORTC QLQ-C30 consists of one single item, hence not a very robust construct. The association could possibly also be due to the relatively low reliability of the Fighting Spirit domain. Also in the Norwegian, Korean and Greek versions of the Mini-MAC, Fighting Spirit has produced lower internal consistency than the other domains [49-51]. This may indicate that Mini-MAC's Fighting Spirit domain can include items not actually measuring this concept or that, due to cultural differences, some items are perceived as irrelevant by patients or hard to interpret.

Several studies have shown a relation between avoidance and poorer HRQL in patients with H&N cancer [25,27,29]. However, we found no such associations but instead that Cognitive Avoidance at one month after treatment start may have a positive effect (not significant) on depression. It has been argued that the effect of an avoidant adjustment response, predominately during the most stressful period with diagnosis and treatment, can be beneficial [21]. Furthermore, there seems to be cultural differences regarding the outcome of an avoidant adjustment response. In the Chinese, Korean as well as the Greek and Italian versions of the Mini-MAC, Cognitive Avoidance is considered to be part of good adjustment [49,50,52,53], while in the Norwegian and English versions Cognitive Avoidance appears to be an indicator for poor adjustment [31,51].

The effect of coping and mental adjustment on survival in cancer patients has been heavily debated during the last decades. Although some studies have found a beneficial effect of a Fighting Spirit response [21,54], some authors have argued that the lack of such findings may be comforting to patients who can not maintain a fighting spirit or a positive attitude [20]. Despite the exclusion of patients with a more advanced disease and lower performance status we found a tendency for the Helpless-Hopeless response to have an adverse impact on survival, results that are in line with Watson et al [19,20]. These findings should however be interpreted with caution due to the relatively small size. One possible explanation for the association between mental adjustment and survival may be that a Helpless-Hopeless response could result in behavioural changes leading to ineffective treatment, e.g. a passive behaviour with failing to come to follow-up visits or neglecting symptoms of recurrence [20,55]. Other possible explanations proposed have been immunological or hormonal [56].

The assessment of mental adjustment has been advocated by several authors, e.g. Matsushita et al, who stress the importance of focussing on patient's individual adjustment styles in order to recognize the specific care needed [10]. It could, therefore, be argued that screening of all laryngeal cancer patients are justified to identify those with maladaptive adjustment responses in order to provide e.g. psychotherapy that may improve adjustment responses [57,58] and possibly even prolong survival [59,60]. Other patients may need more information about their disease or how to get in touch with patient support organisations.

Any instrument used for assessment need to be valid and reliable. A prerequisite for this in PRO instruments is patient input regarding what are relevant items and how they are expressed. Contrary to many other existing PROs the original version of the MAC is based on a great number of patient interviews. However, psychometric evaluations in different languages of the Mini-MAC have demonstrated that the original five factor structure may need to be revised [49-51]. Even though our confirmatory analysis demonstrated agreement with the original five factor solution we found some deviating item loadings.

## Limitations

There were some shortcomings of this study. Firstly, the sample size is quite small. However, laryngeal cancer is a rather uncommon diagnosis, with approximately 200 new cases per year and this is to our knowledge the largest Scandinavian longitudinal study made on mental adjustment and HRQL in patients treated for laryngeal cancer. Another shortcoming might be that excluded patients had a more advanced disease and lower performance status than those included, with a possible underestimation of the prevalence of psychiatric morbidity and use of maladaptive mental adjustment responses. Furthermore, when translating a PRO, a cultural adaptation is also needed to confirm if patients find items to be relevant and intelligible. The Swedish version of the Mini-MAC was evaluated by a group of patients; however, a full-scale cognitive debriefing was never performed. Therefore, to further investigate the content validity of the Mini-MAC in the Swedish laryngeal cancer population, we are currently performing an interview study.

## Conclusions

This study aimed to investigate the relation between mental adjustment to cancer, anxiety, depression and HRQL in laryngeal cancer patients. The findings confirm results previously demonstrated in diverse cancer populations: positive correlations between adjustment responses Helpless-Hopeless and Anxious Preoccupation and anxiety and depression, negative associations with HRQL and a possible relation to poorer survival. This indicates that assessments of mental adjustment should be considered when planning treatment and rehabilitation in patients with laryngeal cancer. Our results also raise the question of a need for further investigating the content validity of the Swedish version of the Mini-MAC Scale. Strengths of this study are its longitudinal design, as well as the focus on patients with laryngeal cancer in which research on mental adjustment and coping has been scarce.

## Competing interests

The authors declare that they have no competing interests.

## Authors' contributions

We hereby certify our personal contribution; all authors were responsible for the design and planning of the study. AR and MJ performed the statistical analysis in collaboration with a statistician. MJ performed the literature search/analysis. All authors were responsible for the data interpretation, participated in preparation of manuscript and read and approved the final manuscript.

## Pre-publication history

The pre-publication history for this paper can be accessed here:

http://www.biomedcentral.com/1471-2407/11/283/prepub
